# Optimizing the Energy Product in Core–Shell Nanoparticle Magnets: General Guidelines and the FePt/CoFe System

**DOI:** 10.3390/ma19112239

**Published:** 2026-05-25

**Authors:** Ioannis Panagiotopoulos, Georgia Basina, Garyfalia Nezou, Alexandros Konstadinidis, Vasileios Alexandrakis, George Hadjipanayis, Vasileios Tzitzios

**Affiliations:** 1 Department of Materials Science and Engineering, University of Ioannina, 45110 Ioannina, Greece; 2 Institute of Nanoscience and Nanotechnology, National Centre for Scientific Research “Demokritos”, 15310 Athens, Greece; g.basina@inn.demokritos.gr (G.B.); g.nezou@inn.demokritos.gr (G.N.); a.konstantinidis@inn.demokritos.gr (A.K.); v.alexandrakis@inn.demokritos.gr (V.A.); v.tzitzios@inn.demokritos.gr (V.T.); 3 College of Engineering, Northeastern University, 360 Huntington Ave, Boston, MA 02115, USA; g.hadjipanayis@northeastern.edu

**Keywords:** permanent magnet, energy product, hard–soft magnetic nanocomposites, core–shell magnetic nanoparticles, magnetization reversal

## Abstract

The optimization of the energy product in permanent magnets presents a complicated multi-parametric problem that encompasses a large variety of intrinsic and microstructural properties. As both high remanent magnetization and coercivity are required, the main concern in optimizing a given material is often how to deal with the trade-off between these two properties. A promising approach is to combine high-anisotropy with high-magnetization phases in chemically synthesized magnetically hard–soft nanoparticles. The magnetization reversal in such systems has been studied by micromagnetics, but most of the solutions are given for a magnetically hard shell surrounding a magnetically soft core, although the inverse configuration may be more accessible from a fabrication perspective and can even help induce tetragonicity in phases such as CoFe. Here we summarize the basic general design rules for such systems, and we present specific calculations for the FePt/CoFe system. Though in larger particles complex reversal modes that are scientifically interesting occur, these are not relevant to the problem of achieving high energy products. Optimal energy products are achieved in small particles in the homogeneous exchange spring regime. Therefore, the optimal size and phase content must be determined under the contradictory requirements of achieving homogeneous reversal and avoiding thermal fluctuations.

## 1. Introduction

The figure-of-merit of a permanent magnet is its energy product, which represents the energy stored in the magnetostatic field it produces per unit volume [1]. A high-energy-product magnet can produce strong magnetostatic fields in a large region without itself being bulky.

Unfortunately, the optimization of the energy product in permanent magnets presents a complicated multi-parametric problem that encompasses a large variety of intrinsic and microstructural properties.

As both high remanent magnetization and coercivity are required, the main concern in optimizing a given material is often how to deal with the trade-off between these two properties.

A rule of thumb can be set by assuming a square *M* vs. *H* hysteresis curve with remanence *M*_R_ and coercivity *H*_C_, leading to the conclusion that the maximum energy product is

(1) $$\begin{array}{c} {{\left(ΒH\right)}_{max}=\mu }_{0}{\left(M_{R}/2\right)}^{2}\;{for\;H}_{C}\geq M_{R}/2,\;\\ {\left(ΒH\right)}_{max}=\mu_{0}\left(M_{R}-H_{c}\right)H_{c}\;{for\;H}_{C}<M_{R}/2\; \end{array}$$

**Figure 1:**
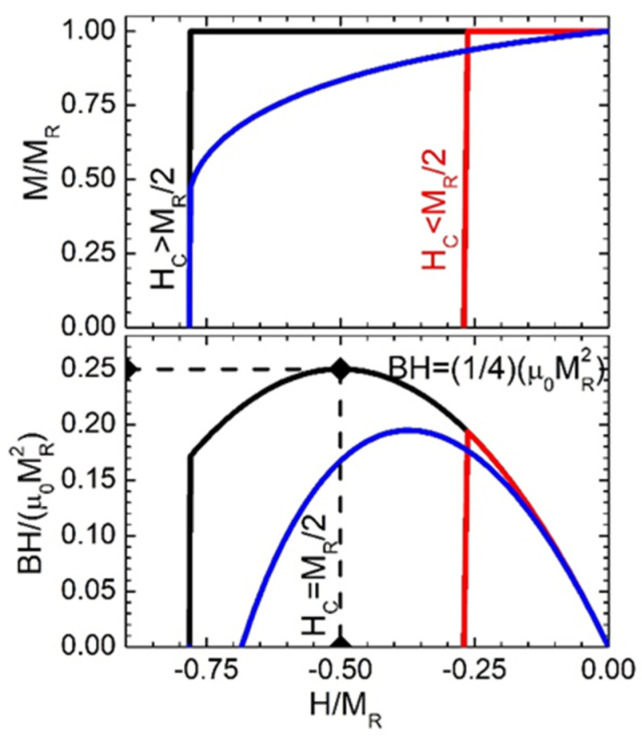
Example demagnetization quadrant *M* vs. *H* curves (**upper panel**) and corresponding *BH* vs. *H* curves (**bottom panel**). For generality the quantities *M* and *H* are normalized in M_R_ and the energy product in ${{\left(ΒH\right)}_{max}=\mu }_{0}M_{R}^{2}\;$ units. The black curves denote a perfect-square loop material with $H_{C}>M_{R}/2$ which reaches the optimal value ${{\left(ΒH\right)}_{max}=0.25\mu }_{0}M_{R}^{2}$. The blue curves give an example of a material with the same coercivity which gives lower ${{\left(ΒH\right)}_{max}=0.20\mu }_{0}M_{R}^{2}$ due to its non-square loop shape. The red curves give an example of a material with a square loop in which ${\left(ΒH\right)}_{max}$ is clearly coercivity limited $H_{C}<M_{R}/2$.

The latter is referred to as the “coercivity limited case”. Such a perfect-square M vs. H hysteresis can be achieved in highly oriented magnets and sets the upper limit for all other cases. A nice example of the use of (Equation (1)) is the optimization of the volume fraction *f* of magnetic particles in pure-shape-anisotropy magnets consisting of elongated magnetic particles bonded in a non-magnetic matrix. In this case $M_{R}=fM_{s}$ and, assuming homogeneous “Stoner–Wohlfarth” switching, $H_{C}=0.5\left(1-f\right)M_{s}$, leading to an optimal ${{\left(ΒH\right)}_{max}=\mu }_{0}M_{S}^{2}/12$ for *f* = 2/3 [2]. Higher values can be obtained if uniaxial magneto-crystalline anisotropy also exists along the same axis as in chemically synthesized cobalt nanorods [3]. Of course, beyond this simple rule of thumb any deviation from squareness in the demagnetization quadrant has a significant effect on the ${\left(ΒH\right)}_{max}$. Figure 1 compares three characteristic cases: (a) a perfect-square loop material with $H_{C}\geq M_{R}/2$, (b) a non-square loop with the same coercivity and (c) a square loop but with coercivity-limited material.

The advent of rare-earth intermetallics marked a revolution in permanent magnet materials thanks to the unique electronic structure of rare-earth elements. These elements exhibit an exceptionally high magnetocrystalline single-ion anisotropy due to the strong spin–orbit coupling of their localized 4f electrons [1]. This intrinsic anisotropy provided a robust mechanism to lock the magnetic moments along preferred crystallographic directions, dramatically enhancing coercivity. However, the saturation magnetization of rare-earth intermetallics themselves is relatively modest. For instance, Nd_2_Fe_14_B with a saturation magnetization of $\mu_{0}M_{s}=1.61\;T$ can give up to ${\left(ΒH\right)}_{max}=516\;kJ/m^{3}$ according to (Equation (1)). Iron with $\mu_{0}M_{s}=2.15\;T$ would give ${\left(ΒH\right)}_{max}=920\;kJ/m^{3}$ if it could be made with sufficiently high coercivity, but this is not feasible because of its low magnetocrystalline anisotropy. Then, an obvious idea is to combine high-magnetization phases with high-anisotropy phases (to provide coercivity) in appropriate nanostructures that result in smooth “one-phase-like” hysteresis with tailor-made $M_{s}$ and ${\;H}_{C}$ [4,5,6,7,8,9].

These nanocomposites have been achieved in melt-spun [10,11,12], mechanically alloyed [13,14,15] and multilayered thin-film structures [8]. One may argue that even Nd_2_Fe_14_B is a naturally made multilayered structure consisting of alternating crystallographic planes of Nd and Fe in atomic ratios that optimize anisotropy and magnetization. However, the outlook of discovering optimized ternary or more complex phases’ properties seems poor, although recently a high-entropy alloy approach is being explored based on creating artificial multielements as building blocks to discover and stabilize novel microstructures [16].

Another possible geometry towards hard–soft nanocomposites with well-controlled nanostructures is based on the chemical synthesis of hard–soft nanoparticles [17] preferentially with core–shell morphology [18,19,20,21,22,23]. One can note that there is less experimental work on fully metallic systems [22,23]. As a general guideline, since the optimal composition boils down to finding the maximum soft-phase content which increases the magnetization without a detrimental loss of coercivity, it is expected that the optimized compositions would be in the vicinity of those compositions leading to “coercivity-limited” *BH*_max_.

## 2. Can Simple Design Rules Be Set up for the Optimization of Nanocomposite Permanent Magnets?

As regards remanence, a simple rule-of-thumb can be used, as its value depends on the easy axis distribution and the magnetic-material packing fraction *f* and scales as

(2) $$M_{R}=f\langle cos\theta \rangle M_{S}$$

where θ is the angle between the anisotropy axis and the field. This equation is valid for materials with uniaxial anisotropy, which are the typical materials of choice for permanent magnet applications. In an “isotropic material” with a completely random distribution of easy axes, $\langle cos\theta \rangle =1/2$. Thus, the attainable energy product in isotropic magnets is only ¼ of that of highly oriented materials, but the required coercivity is also lower according to Equation (1). We must note here that in nanomaterials with strong intergrain coupling, “remanence enhancement” above the values of Equation (2) may be observed. This enhancement has been observed both in single-phase and nanocomposite materials consisting of a fine mixture of soft and hard phases [24]. In a typical permanent magnet manufacturing process, both *f* and $\langle cos\theta \rangle$ are optimized: to obtain a compact with maximum remanent magnetization, the metallic powders are aligned and pressed under a field such that the easy axes of magnetization of the powders are parallel. Then, sintering is carried out either in vacuum, inert gas atmospheres or reducing atmospheres to achieve the highest density possible without significant loss of coercivity [25].

For magnets made by compacting chemically synthesized magnetic particles, let us note that the theoretical packing fraction limit for cylindrical particles is close to f = 0.9 [3], while for spheres f = 0.74 [26]. Thus, in principle, according to (Equation (2)), cylindrical particles can achieve 33% higher (*BH*)_max_ compared to spherical ones.

The situation is much more complex when it comes to predicting the coercivity and the loop shape in general. Any deviation from squareness, increased permeability or discontinuous jumps at the demagnetization quadrant is detrimental to the energy product. These characteristics are very sensitively dependent on the exact microstructure characteristics, making the establishment of hard-and-fast material design rules difficult.

In the case of hard–soft nanocomposites, again, the magnetization is simply equal to the volume average of the magnetizations of the two phases, but this is not necessarily true for other quantities, especially the coercivity [27]. The typical magnetization reversal proceeds by nucleation in the soft phase and propagation to the hard phase. If, after nucleation, propagation does not occur, the coercive field is defined by the propagation. The critical fields depend, apart from the parameters of each phase, on the microstructure and interfacial coupling between the two phases.

The mechanisms of nucleation and propagation are more easily separated and modeled in multilayer [28] or cylindrical structures [29] that can be reduced to 1D models [30]. In some cases, even macro-spin models may give a good approximation [31].

It is often considered that if the two phases are rigidly coupled, and the dimensions are below a critical thickness, the composite system is characterized by the averaged magnetic properties of the two layers, yielding a nucleation field [8,31,32]:

(3) $$H_{N}=\frac{2\left(p_{H}K_{H}+p_{S}K_{S}\right)}{p_{H}M_{H}+p_{S}M_{S}}$$

where $p_{H},p_{S}$ are the volume fractions of the hard and soft phases respectively. However, the validity of this simple composite-material rule is obviously limited to very fine dispersions of the two phases. In general, the phases must be suitably dispersed and mutually exchange-coupled, to avoid independent switching of the two phases giving rise to what is termed as “stepped”, “shouldered” or “wasp-waist-like” hysteresis and maintain sufficiently high coercivity. The nucleation scales with the dimension *L* of the soft phase as 1/*L*^2^, while propagation depends on the difference in domain wall energies between the two phases [33]. The main criterion could be set by the distance to the nearest hard region [27], keeping in mind that the critical dimensions are in the nm range, following the magnitude of typical magnetic characteristic lengths as exchange lengths (*L*_ex_) and domain wall widths.

In nanoparticles the reversal mechanism is more complex. Even in single-domain and single-phase materials, above a critical diameter, an inhomogeneous reversal (“curling” mode) becomes favorable because it follows a “pole-avoidance” path that reduces the magnetostatic contribution by the formation of a vortex-like intermediate state [34,35]. While the homogeneous “Stoner–Wohlfarth” reversal for spherical particles predicts a coercive field of $H_{C}^{SW}=2K/{\mu_{0}M}_{s}$, curling occurs at a field

(4) $$H_{C}^{Curl}=\frac{2K}{{\mu_{0}M}_{s}}-\frac{1}{3}M_{S}+\frac{8.666\;A_{ex}}{{\mu_{0}M}_{s}R^{2}}$$

where Aex is the exchange stiffness. The curling becomes favorable for radii

(5) $$R>R_{c}=3.6L_{ex},\;L_{ex}=\sqrt{2A_{ex}/\left(\mu_{o}M_{S}^{2}\right)}$$

When it comes to spherical soft/hard particles, two separate cases can be distinguished:

When a soft-magnetic core is surrounded by an infinitely hard-magnetic phase, nucleation is realized by a mode called “bulging,” which is considered to have radial angular symmetry of the coherent mode [36]. If the demagnetizing fields are accurately taken into account, the bulging mode ceases to be an eigenmode for nucleation. At small core sizes, the nucleation of magnetic reversal proceeds via a modified bulging mode, where the transverse component of the magnetization is only semi-coherent in direction and the nucleation field contains a contribution from self-demagnetization [37]. In this case the coercivity has a similar dependence on (Equation (3))

(6) $$H_{C}^{mbulg}=\frac{2K}{{\mu_{0}M}_{s}}-\frac{1}{3}\left(M_{S}-M_{H}\right)+\frac{19.74A_{ex}}{{\mu_{0}M}_{s}R^{2}}+0.975M_{s}$$

For large core sizes, the modified curling mode, where the magnetization configuration is vortex-like, is favored at $R>R_{c}=3.25L_{ex}$. It has a vanishing demagnetizing field because of the flux closure.

(7) $$H_{C}^{mcurl}=\frac{2K}{{\mu_{0}M}_{s}}-\frac{1}{3}\left(M_{S}-M_{H}\right)+\frac{40.382A_{ex}}{{\mu_{0}M}_{s}R^{2}}$$

**Figure 2:**
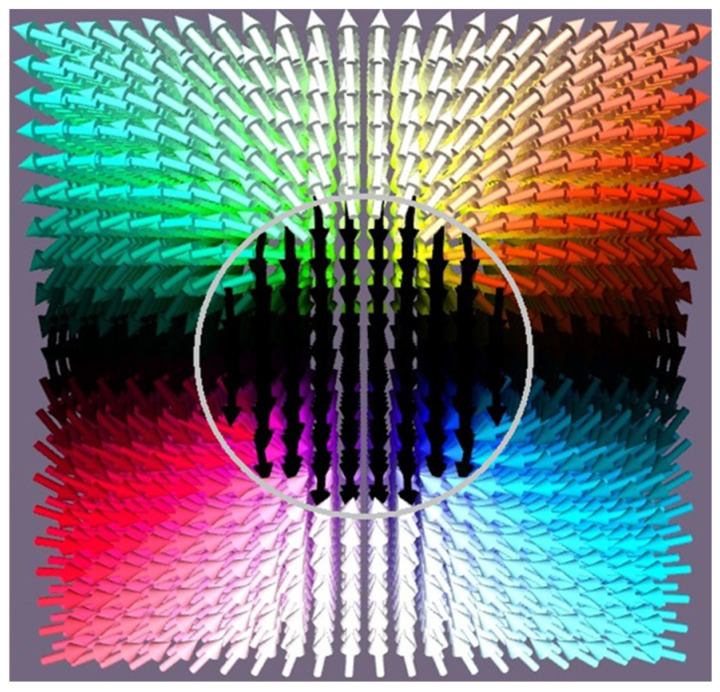
Typical stray field created around a hard-phase sphere homogeneously magnetized upwards. The color code indicates the direction of the vectors: White for upwards, black for downwards, red to the left, blue to the right and in between colors for other directions.

Analytical expressions for the case of a hard-magnetic core surrounded by a soft-magnetic shell are scarce and mostly given for cylindrical structures [37]. The dipolar field generated from the core is expected to impact the magnetization reversal [38], since in the equatorial region it is directly opposite to the initial magnetization direction (Figure 2). Its inhomogeneous nature is expected to favor the formation of inhomogeneous modes of reversal, such as curling and flower modes in the surrounding shells, although these effects would manifest only for sufficiently thick shells.

## 3. The FePt/CoFe Core/Shell System

Here we focus on FePt/CoFe as an example system using micromagnetic simulations performed by the mumax3 finite difference micromagnetic simulation program [39,40,41]. Numerical micromagnetics is a well-established continuous medium theory in which the magnetic state of a particular magnetic body is described by the spatial dependence of the magnetization vector that has a length equal to the saturation magnetization *M*_S_ and a direction which is a function of position within the material m(r). The presence of local minima in which the magnetic state can be trapped yields the well-known hysteretic effects that are typically observed in magnets. Monodisperse FePt nanoparticles can be synthesized by chemical liquid-phase high-temperature reduction approaches, in high-boiling-point organic solvents with the presence of a variety of capping agents and have been extensively studied since the seminal work of Sun [42]. The preparation can be followed by annealing to induce L1_0_ chemical ordering and therefore high anisotropy. Using coercivity vs. pulse width data, a high uniaxial anisotropy of 5.9 × 10^6^ J/m^3^ was estimated for 4 nm particles. The use of bismuth additives in the reaction permits a direct one-step liquid-phase chemical approach [43,44,45,46].

The Co-Fe alloys, typically used as soft-magnetic materials, give a record magnetization of 1.95 MA/m for the composition Co_35_Fe_65_. High saturation values are, in principle (according to the Slater–Pauling curve), obtained up to the equiatomic composition, though the numbers reported in the literature vary depending on the partial chemical ordering, exact composition, impurities and existence of dead or oxide layers in films and particles. Liu and coworkers give for the near equiatomic (Fe_53_Co_47_) films Ms = 1.35 MA/m [47]. For Fe_52_Co_48_, 1.9 MA/m is used [48], and for equiatomic FeCo nanowires, 1.71 MA/m [49]. Interestingly, FeCo nanoalloys can be chemically synthesized in liquid-phase reactions with the ability to control their sizes in the nanosize regime [50]. Interestingly, CoFe by itself could make a high-performance magnet since it is predicted that by chemical ordering to a bct phase with optimized parameter of c/a = 1.20–1.25, it can achieve a high value of Ku ≈ 10 MJ/m^3^, which is higher than that of FePt [51]. However, this has been achieved in thin films mainly by taking advantage of epitaxial strain in carefully designed multilayer systems [52,53,54]. An equivalent approach in nanoparticle systems is to grow FeCo shells on AuCu cores thermally treated to induce transformation to the tetragonal L1_0_ phase [55]. The AuCu was chosen due to lattice matching. Above a critical shell thickness (3.17 nm), the FeCo shell returns to the bcc structure via strain relaxation. This is scientifically interesting but as a permanent magnet its (*BH*)_max_ is limited by the low volume fraction of the magnetic phase, leading to low remanence. For instance, assuming close-packed spherical particles with a 3.5 nm magnetic shell on a 10 nm AuCu core, the volume fraction will be 0.48. For a saturation magnetization of 1.8 MA/m, the maximum achievable energy product is 240 kJ/m^3^. In comparison, using single-phase FePt nanoparticles with magnetization of just 1.1MA/m, the maximum achievable energy product is 210 kJ/m^3^. Ideally, one should seek to achieve the same kind of templated growth on a magnetic L1_0_ phase with the appropriate lattice constant.

For the hard phase, the following parameters of the equiatomic chemically ordered FePt (with the tetragonal L1_0_ structure) are used: saturation magnetization *M*_S_ = 1.1 MA/m, uniaxial anisotropy *K*_mc_= 4.9 MJ/m^3^ and exchange stiffness *A*_ex_ = 10 pJ/m [42,56,57,58]. For the CoFe different values are given depending on the exact composition and chemical ordering. In this study the following were assumed: *M*_S_ = 1.8 MA/m [55], *K*_mc_ = 10 kJ/m^3^, and *A*_ex_ = 25 pJ/m [48,59,60]. A small misalignment of 1.0 deg with the applied field was introduced to avoid numerical errors that could arise in the case where the axes of the magnetocrystalline and applied field coincide.

**Figure 3:**
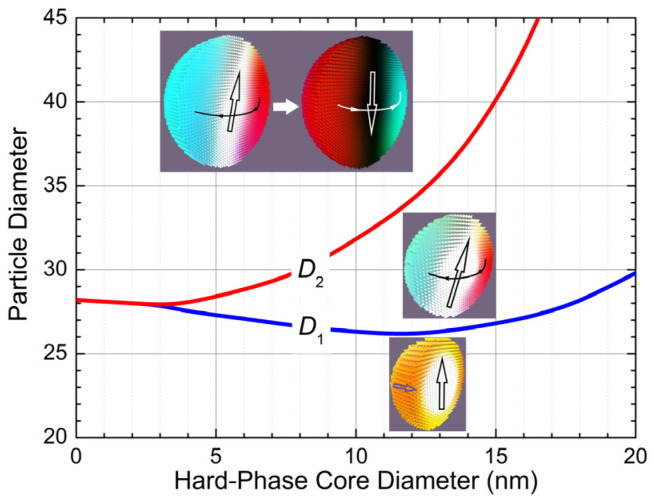
Diagram of different modes of reversal occurring as a function of the hard-phase core and total diameter of core–shell nanoparticles. Below a critical thickness D_1_, the reversal proceeds by a homogeneous canting of the soft shell. Above D_1_ the reversal proceeds by a curling mode which also includes the formation of a reversed vortex above D_2_. The insets show characteristic spin configurations for each region. Color code: White spin up, black spin down, red spin outwards, blue spin inwards, yellow spin to the right.

In Figure 3, the various types of pathways of the magnetization reversal are summarized as a function of the magnetically hard-core diameter and total particle diameter. We can distinguish three qualitatively different regimes: For small diameters the reversal proceeds by a homogeneous canting of the soft shell, which increases with the applied reversed field until this state is destabilized and leads to an abrupt reversal to the homogenous reversed state ($m=M/M_{s}=-1$). Above a critical thickness D_1_, which depends weakly on the hard-phase diameter (ranging between 28 nm and 30 nm), the reversal proceeds by a curling mode. The axis of the vortex tilts slightly with the applied reversed field until the state is destabilized and an abrupt reversal to the homogenous reversed state (m = −1) occurs. Above a second critical thickness D_2_, which depends strongly on the hard-phase diameter, the reversal proceeds by a curling mode in which a reversed-vortex state is also formed. The axis of the vortex tilts slightly with the applied reversed field until the vortex is reversed. This reversed vortex evolves gradually by spin-canting towards the z-axis to the final homogenous reversed state (m = −1).

**Figure 4:**
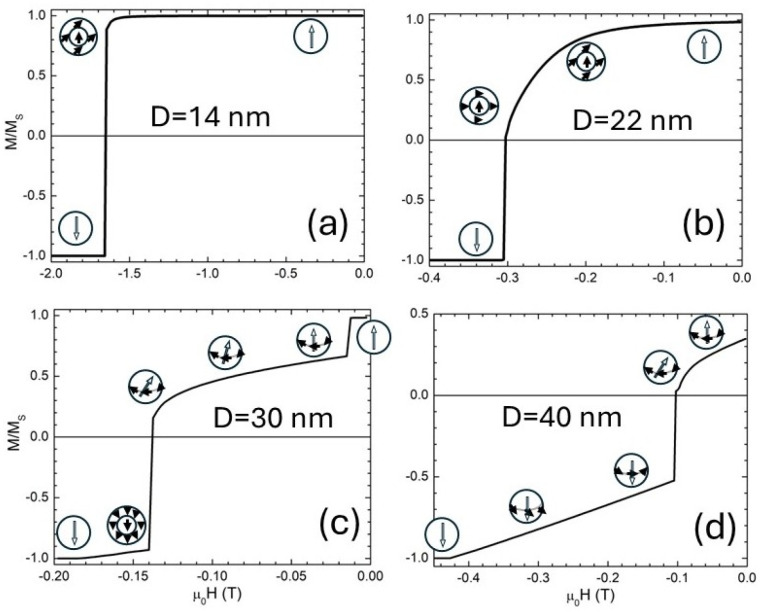
Typical demagnetization curves corresponding to three cases of Figure 3. The insets show the corresponding spin configurations along the demagnetization curves. In all cases the magnetic hard-phase diameter is 10 nm. (**a**) Particle diameter 14 nm: The reversal starts with a small homogeneous canting of the soft shell, which destabilizes the particle and leads to an abrupt reversal. (**b**) Particle diameter 22 nm: The reversal proceeds by a gradual homogeneous canting of the soft shell and loss of squareness, which increases with the applied reversed field until this state is destabilized, followed by an abrupt full reversal. (**c**) Particle diameter 30 nm: The reversal proceeds by a curling mode, which includes the formation of a vortex that is destabilized, and follows an abrupt reversal to a reversed state. (**d**) Particle diameter 40 nm: The reversal proceeds by the formation of a vortex, which is reversed and gradually tilts towards the completely reversed state.

Typical corresponding demagnetization curves for four cases (points on Figure 3) are shown in Figure 4. A hard-phase core of 10 nm with different diameters has been selected. A rapid loss of coercivity and squareness is observed with particle size, even for the homogeneous canting regime. The existence of non-homogeneous modes of reversal leads to a severe reduction in the magnetization in the demagnetization quadrant of the hysteresis loop, which is deleterious to the (*BH*)_max_. Although these modes are scientifically interesting (and even lead to the existence of topologically non-trivial configurations [61]), for permanent magnet applications the particle size should be restricted below 26 nm.

These simulations further show that Equation (3) holds for very small diameters. Using Equation (3) one can calculate, for the given FePt and CoFe phase parameters, a maximum (BH_max_) = 505 kJ/m^3^ achieved for hard-phase content p_H_ = 0.12 (the compositions with p_H_ < 0.126 being coercivity limited). In other words, the hard-phase core could be almost half (equal to 0.4936) of the total diameter.

**Figure 5:**
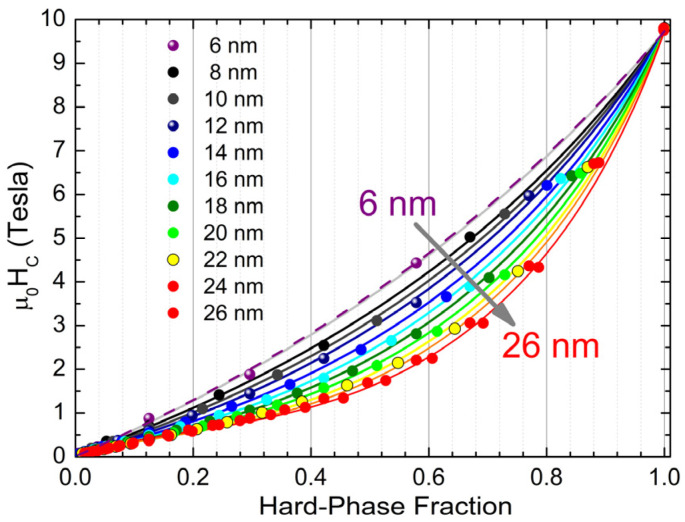
Coercivity vs. hard-phase fraction for core/shell nanoparticles of different diameters (indicated in nm). The simple composite-material rule implied by Equation (3) (dashed line) can only describe the 6 nm particles. For the higher diameters, strong deviations are observed that can be phenomenologically described by Equation (8) (continuous lines).

A comparison of the coercivities derived by our simulations to the predictions of Equation (3) is presented in Figure 5. Only the D = 6 nm case is close to the predictions of Equation (3).

For the rest of the cases the data can be phenomenologically fitted by assuming volume fraction corrected by an additional non-linear term that increases with diameter. We have used the form for the effective volume fraction:

(8) $$p_{H}^{eff}=p_{H}+{a\cdot p}_{H}^{n}ln\left(p_{H}\right)$$

Note that the data (as well as Equation (8)) imply that for either $p_{H}=0\;or\;1$ the Stoner–Wohlfarth prediction holds $\left(as\;p_{H}^{eff}=p_{H}\right)$. Thus, it is the two-phase nature of the particles that enhances the deviations from the simple Stoner–Wohlfarth prediction, which is reasonable due to the spherical geometry with an inhomogeneous radial profile. For the data of Figure 5, the non-linear part scales with the diameter as a=(D−16.1 nm)/15.3 nm, while the exponent *n* varies between *n* = 1.4 and n = 1.8.

On the other hand, the paramagnetic size for the hard phase can be calculated to *D*_p_ = 3.2 nm. Therefore, sizes must be well above 3.2 nm to avoid a coercivity reduction on the order of ${\left(D_{p}/D\right)}^{3/2}$. In conclusion, zero-temperature calculations cannot be safely used for the prediction of coercivity and energy products. Even for 26 nm the expected thermal reduction in coercivity is close to 4%, meaning that in this range of diameters the temperature effects cannot be ignored. The optimal size and phase content must be determined under the conflicting requirements of having a particle small enough to achieve a homogeneous reversal and large enough to avoid thermal fluctuations.

To take into account thermal effects, a reduction in coercivity on the order of $1-\sqrt{T/T_{B}}$ (at T = 300 K) was assumed and the blocking temperature was calculated using the relation $25k_{B}T_{B}=V_{H}K_{H}+V_{S}K_{S}$. This simple textbook formula is derived assuming an Arrhenius law with a fixed attempt frequency of 1 GHz [1] and measurement time of 60 s. For measurement times of one hour or one day, $29k_{B}T_{B}$ and $32k_{B}T_{B}$ should be used respectively. In fact, the results of mumax3 simulations at finite temperature [39] are compatible with a field-dependent attempt frequency as analyzed in [62]. This model equivalently gives a sharply peaked attempt frequency on the order of 1 GHz for applied reversed fields close to the anisotropy field. Let us also note that thermal fluctuations induce more homogeneous modes of reversal compared to an applied field. This principle, which permitted the optimization of exchange spring recording media [63], implies that temperature is not expected to affect the loop squareness unless a broad particle size distribution is present.

**Figure 6:**
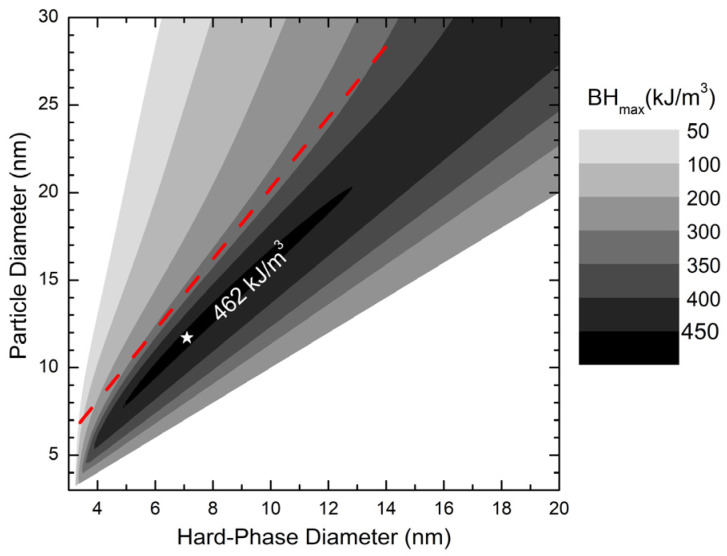
Energy product contour plot as a function of the hard-phase diameter core and total particle diameter. The star indicates the point of optimum hard-phase and overall particle diameter combination which leads to an energy product of 462 kJ/m^3^. The dashed red line shows the locus of the maximum energy product according to Equation (3) and without taking into account the temperature effects. Taking these extra factors into account, a slightly larger hard-phase content is needed.

Taking all these factors into account (Figure 6), the maximum energy product of 462 kJ/m^3^ is obtained for a particle of 11.7 nm with an FePt core of 7.1 nm (p_H_ = 0.223).

The calculations presented here have been performed using an interfacial coupling as strong as 14 mJ/m^2^. However, despite the fact that good interface matching is desirable in the hope of promoting CoFe tetragonality, in a core–shell system, reduction in interfacial exchange is probable. This would affect the results and the diagram of Figure 3. Focusing on the optimal design, we have studied the effect of interfacial coupling in a 12 nm particle with a 7 nm core. The loops become obviously stepped (two-phase-like) if the exchange is reduced below 3 mJ/m^2^. At 3 mJ/m^2^ the loops are smooth but the coercivity is reduced by 20% with respect to full coupling. Above 10 mJ/m^2^ the coercivity and the energy product are practically unaffected.

Another question that should be addressed is the effect of dipolar interactions in permanent magnets consisting of dense particle assemblies. As noted in reference [2], at high packing fractions the demagnetization effects depend mainly on the overall shape of the magnet. In industrial product specifications the magnets are characterized by closed-flux measurements that correspond to zero demagnetization, in which case no appreciable difference from the isolated loop is expected. The demagnetizing effects are taken into account by the load line, which determines the working point [35]. In terms of individual particle interactions, the expected effect is as follows: Local dipole stray fields are magnetizing in lines of particles along the magnetization direction and demagnetizing in perpendicular planes. As a result, there is a tendency to have correlated reversal in lines of particles at fields weaker compared to that of the isolated particle. However, these reversals tend to stabilize nearby particles, which will tend to reverse at higher fields compared to that of the isolated particle. Therefore, in the limit of a system consisting of a huge number of particles we expect a broadening of the demagnetization curve (loss of coercivity squareness) which would be proportional to the demagnetizing factor (overall shape) of the magnet.

**Figure 7:**
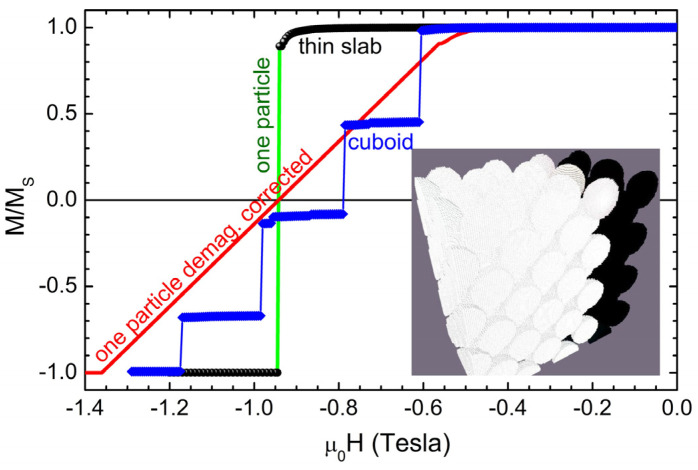
Demagnetization curves of particle arrays in different agglomerate shapes. Black circles: Thin slab, 504 × 108 × 504 nm^3^. Blue diamonds: Cuboid, 504 × 468 × 504 nm^3^. Continuous green line: One isolated particle. Continuous red line: One isolated particle corrected for a demagnetization factor *N* = 0.32. The total sizes reported are made of periodic copies of a system, 72 × 36 × 72 nm^3^, which includes 128 particles of diameter 12 nm. The inset shows a typical micromagnetic configuration of the 72 × 36 × 72 nm^3^ in the cuboid, at *μ*_0_*H* = −65 T, *M*/*M*_s_ = 0.45: each of the constituent particles stays in homogeneous positive (white)- or negative (black)-magnetization state. The tendency to reverse in regions along the vertical field direction becomes visible.

Some indicative simulations in systems consisting of 128 particles arranged in fcc-type close-packed arrays are included in Figure 7. The simulated cell is 72 × 36 × 72 nm^3^ and includes 32 cubic unit cells of dimensions 18 × 18 × 18 nm^3^. The particles have a total diameter of 12 nm (and a 7 nm hard-phase core), which gives a distance of 0.73 nm between them. To study the effect of demagnetizing fields in larger arrays of adjustable demagnetization, periodic boundary conditions are imposed, and furthermore, several periodic images (copies of the system) in each direction are considered. We present results on two representative cases: (i) Thin slab, 504 × 108 × 504 nm^3^, which has a negligible demagnetization factor $N_{z}\approx 0.08$ along the field direction. (ii) Cuboid, 504 × 468 × 504 nm^3^, which has a demagnetization factor along the field direction $N_{z}\approx 0.32$. For the thin slab, as a result of the low demagnetizing effect, the demagnetization curve coincides with that of the isolated particle. For the cuboid a series of jumps corresponding to simultaneous reversal of groups of particles is observed, which follows the trend of the demagnetization-corrected curve of a single particle. The stepped curves are a result of the limited number of particles (and switching events thereof) in the simulation. In the large particle limit, the step of each switching event will become negligible with respect to the total magnetization change and the curve will become smoother.

## 4. Discussion and Conclusions

The idea to create high-performance magnets by fabricating nanocomposites consisting of high-magnetization and high-anisotropy phases dates back several decades [4] but has attracted the uninterrupted interest of the scientific community, which has intensified lately due to the recent instabilities in rare-earth supply [64]. The interest in hierarchical bottom-up production of magnetic materials existed before the rare-earth supply problem [9] and continues to date in view of a large variety of applications [65]. Methods using adjustable high-temperature continuous-flow reactors can be used to scale up the process to industrial production [66]. The rich variety of possible existing systems and the complex reversal mechanisms they may exhibit make it seem impossible, at first sight, to establish hard-and-fast design rules. However, keeping in mind that deviation from loop squareness is detrimental to the (*BH*)_max_, this makes it obvious that optimized systems should consist of small entities that are characterized by homogeneous reversal modes. More complex modes are scientifically very interesting and may include topological configurations, but they are not relevant to the problem of achieving high energy products. In short, the optimal size and phase content must be determined under the contradictory requirements of achieving homogeneous reversal and avoiding thermal fluctuations. One should keep in mind that any reduction in magnetic packing fraction, such as the use of bonding materials, reduces the energy product by the square of the packing fraction. In this respect spherical nanoparticles are disadvantageous as in principle they can achieve a maximum packing fraction of f = 0.74. In contrast, in needle-like particles, f = 0.9 is achievable. Aligning in a magnetic field is straightforward and, in principle, can quadruple the energy product, so it is recommended. In short, we can summarize the rules as system-independent, i.e., achieving a high degree of alignment and packing fraction, and system-dependent. For the latter, a first approximation can be done using Equation (3), assuming that the reversal is homogeneous, to estimate the optimal hard/soft-phase ratio and take into account superparamagnetic effects to determine the optimal particle size. However, the answer will be only approximate and micromagnetic simulations should be used to extend the accuracy and validity of Equation (3), possibly with the use of an effective volume fraction. Here we have used micromagnetic simulations to analyze the FePt/CoFe hard-magnetic core/soft-magnetic shell system. The same method can be applied to any choice of materials and geometry.

## Data Availability

The original contributions presented in this study are included in the article. Further inquiries can be directed to the corresponding author.

## Abbreviations

The following abbreviations are used in this manuscript:

(*BH*)_max_	Maxiumum Energy product
